# In vitro ruminal fermentation, core nutrient, fatty acids and mineral matter of pennyroyal (*Mentha pulegium* L.) herbage at different phenological stages

**DOI:** 10.1002/vms3.1397

**Published:** 2024-03-07

**Authors:** Kanber Kara, Sena Yilmaz, Berrin Kocaoğlu Güçlü, Seyrani Demir

**Affiliations:** ^1^ Faculty of Veterinary Medicine Department of Animal Nutrition and Nutritional Diseases, Erciyes University Kayseri Türkiye

**Keywords:** α‐linolenic acid, fatty acid, linoleic acid, pennyroyal, phenological stage

## Abstract

**Background:**

In ruminants, fibrous feedstuffs must be included in the ration to ensure normal rumen physiology and to prevent the occurrence of rumen‐related metabolic diseases. In addition to being a source of fibrous feedstuffs, they contain energy depending on the level of digestion and protein, minerals, fatty acids, minerals, and secondary compounds.

**Objectives:**

This study aimed to determine the nutrient matter, fatty acid, mineral and in vitro rumen fermentation values of the pennyroyal (*Mentha pulegium* L.) plant.

**Methods:**

The pennyroyal plant samples were collected at different phenological stages (vegetative, full flowering, and seed bulking) from the natural meadow. The samples were analysed for core nutrients, condensed tannins, minerals, fatty acids, and in vitro ruminal fermentation parameters.

**Results:**

The calcium (Ca) and iron (Fe) contents and in vitro ruminal fermentation parameters (total gas production and methane production, organic matter digestion (OMd), and the ammonia‐nitrogen) decreased with increasing phenological stage (*p* < 0.05). The percentages of linoleic, α‐linolenic, ω‐3, ω‐6 and polyunsaturated fatty (PUFA) acids of the pennyroyal plant linearly increased with the phenological stages (*p* < 0.05). However, butyric acid (BA) concentration in the in vitro ruminal fermentation fluid in the full flowering stage was lower than that of other stages (*p* < 0.05).

**Conclusions:**

Pennyroyal plant is a functional plant in terms of high values of ether extract (EE), α‐linolenic acid, linoleic acid, ∑ω‐3 fatty acids, Ca, Fe and Zn contents. For this plant to be used as animal feed, the stage when it has the highest values for Ca, Mg, S and Zn minerals and in vitro OMd were vegetative and full flowering. The stage with good potential as animal feed for ∑ω‐3 and ∑ω‐6 fatty acids and core nutrients (CP and EE) is the seed bulking stage.

## Graphical abstract

With the phenological stage of pennyroyal, Ca, Mg and S macro minerals, Zn and Fe micro minerals, myristic and palmitic acids and in vitro ruminal total gas, methane, ammonia‐nitrogen and organic matter digestion generally decreased. However, CP, fibrous compounds, nonfibre carbohydrates, condensed tannin and metabolic energy values of plant varied for increasing stages. When the plant reached the seed setting period, its EE content was very high as for forage and its ω‐6 linoleic and ω‐3 α‐linolenic acids contents increased.

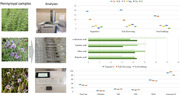

## 1 INTRODUCTION

Fibrous feedstuffs are used as a dietary fibre source due to their effects on buffering acidity in rumen fermentation, rumination, animal product quality and animal health (NRC, 2001). The quality of the forage is affected by many factors. The type of forage plant, the phenological stage in which the plant is harvested, climatic conditions, the characteristics of the soil at which the plant grows and the way of storage are among these factors. Due to the importance of sustainable animal husbandry in recent years, the search for a perennial alternative and functional forage that can remain green for most of the year, taking into account global warming, is continued by researchers (Ersahince and Kara, 2017; Kara et al., 2018; Kara, 2021). Forages grown in areas with high annual precipitation are of good quality, whereas forages grown in areas with arid or semi‐arid climatic conditions are only green in spring and early summer. Towards the end of the summer, some of the forage plants turn yellow and dry (Kara et al., 2018). Harvest time in the phenological stage of the forage plant is an important factor that plays a role in the quality of the forage. The progressing of phenological stage decreases forage quality (Kara, 2021). Pennyroyal (*Mentha pulegium* L.) is among the *Mentha* species in the *Lamiaceae* family. These species are also known by the name pennyroyal. It is found in natural vegetation in many regions of the Northern Hemisphere, including Türkiye (Ölmez & Makav, 2021). The green part of the pennyroyal plant contains terpene compounds such as pulegone, menthone, piperitone, piperitenone and isomenthon (Teixeira et al., 2012). The essential oils obtained from the pennyroyal plant and the dry parts of the plant have been traditionally used as both antiseptic and antibacterial in the treatment of digestive system diseases and respiratory system diseases, as well as having analgesic, antiemetic, and antioxidant effects (Boukhebti et al., 2011). *Mentha arvensis*, *M. longifolia* and *M. spicata*, which are among the *Mentha* species, are used in the treatment of vomiting and nausea, while *Mentha pulegium* is used in the production of synthetic menthol; *Mentha piperata* in the pharmaceutical and apparel field; *Mentha spicata* leaves are used in the treatment of indigestion and rheumatic diseases; *Mentha longifolia* is used in the treatment of rheumatic pain and circulatory system diseases (Sharma, 2004). This study aims to investigate the in vitro digestion of the pennyroyal plant, the changes in core nutrient and fatty acid compositions at different phenological stages and to use of the pennyroyal plant as an alternative roughage source in ruminant nutrition. Today, when the effect of drought is evident, alternative forage crops that are compatible with arid climatic conditions, suitable for harvesting, remain green throughout the year, and even contain functional compounds should be investigated (Kara et al., 2022; Pastorelli et al., 2022; Pirasteh‐Anosheh et al., 2021). The study hypothesizes that the in vitro digestion and nutrient level of the pennyroyal plant as a forage varies according to the phenological period.

## 2 MATERIAL AND METHOD

### 2.1 The plant samples

The pennyroyal (*Mentha pulegium* L.) plant samples were collected at different phenological stages from natural meadows of Kayseri province, Türkiye. Kayseri province has arid conditions and desert‐like steppe vegetation due to temperature and rainfall (Altin et al., 2012). The monthly average total precipitation amount was 36.1 mm for June, 14.2 mm for July and 12.7 mm for August. Annual precipitation of Kayseri was 391.1 mm. The samples of phenological stages were collected at different times according to the plant growth period. Sampling times were June for vegetative, July for full flowering and August for seed bulking. The region where the samples were taken is in summer in the months specified. Samples included all aerial parts of the plant (leaf, stem, or bud flower). A random sampling method was used from native grassland. Cutting was manually performed at 1 cm above the soil, in the middle of a 10 m^2^ delimited area. The collection of plant samples was done manually with shears about 1 cm above the soil. Random sampling was done from the field for plant collection, and approximately 8 kg of samples were collected for each phenological period.

### 2.2 Chemical analyses

The herbage samples were manually cut into 2–3 cm lengths with a knife. Subsequently, herbage samples were dried for 48 h at 60°C in an oven (Binder, Germany), and the dry matter (DM) content of herbages was calculated. Dried‐milled herbage samples (a diameter of 1 mm) (Laboratory type grinder, IKA MF 10.2, IKA Werke, Germany) were analysed for diethyl ether extract (EE), crude protein (CP), ash (AOAC, 1995), neutral detergent fibre and acid detergent fibre without ash content (NDFom and ADFom, respectively) (Van‐Soest et al., 1991) and nonfibrous carbohydrate (NFC) (NRC, 2001). Hemicellulose (HC) content was calculated from difference between NDFom and ADFom (HC = NDFom – ADFom). The analyses of all these chemical compositions were conducted in triplicate. The analysis was repeated when the difference between the results of repeated analyses was greater than 5%.

For mineral analysis, plant samples were first melted in a platinum crucible. From the sample to be combusted, 1.3 g is weighed into the platinum crucible, and 13 g of the mixture of lithium tetraborate + lithium metaborate (67% + 33%) is weighed. A very small amount of lithium iodide is added to the mixture to prevent the sample from sticking to the platinum crucible, mixed with a spatula and placed in the fusion device (Claisse LeNeo Fusion Instrument, Malvern Panalytical Ltd, Malvern, United Kingdom) and the sample becomes glassy at 1065°C for 25 min and becomes ready for analysis. Minerals were read a XRF spectrometer (PANalytical Axios Advanced, Malvern Panalytical Ltd, Malvern, United Kingdom) in triplicate.

For fatty acids analysis, after methylation with methanolic potassium hydroxide and precipitation with sulphuric acid, methylated free fatty acids were taken into n‐hexane (Wang et al., 2015). Methylated bound and free fatty acids were detected in a gas chromatograph with flame‐ionization detection (GC‐FID, Thermo Scientific, USA). The analysis procedure was according to Kara (2020). A column of the fatty acid methyl esters (FAMEs) was used for the separation of fatty acid (cyanopropylphenyl‐based phase, length: 60 m, internal diameter: 0.25 mm, film: 0.25 μm and maximum temperature 250–260°C; Thermo Scientific TRACE™, TR‐FAME GC Columns, catalog number: 260M153P, USA). Peak identification of each FAME was used in a standard mixture solution of a commercial FAME in dichloromethane (Chem‐Lab, catalog number: CL.40.13093,0001, Zedelgem, Belgium) to define retention times and areas

Condensed tannins contents of pennyroyal herbage’ samples were determined by Makkar et al.’s (1995) modified method. The 0.05 g of sample is taken into 15 mL polypropylene falcon tubes and 6 mL of n‐butanol, HCl and FeSO_4_ (for 100 mL of solution: 95 mL of n‐butanol, 5 mL of 37% HCl, 0.05 g of FeSO_4_) solution are added and vortexed. The prepared samples were incubated for 1 h in the oven at 100°C. The supernatants of the centrifuged samples were read at 550 nm in a UV‐VIS spectrophotometer (UviLine 8100, SI Analytics; Mainz, Germany) and total condensed tannin (TCT) content was calculated. To determine the bound condensed tannins (BCT), 0.05 dried sample was weighed into 15 mL tubes and 5 mL of acetone (70%, distilled water) was added. Vortexed tubes were kept at room temperature for 24 hours. Tubes were centrifuged (Nüve NF 400R, Ankara, Türkiye) at 2750 × *g* for 5 min under 21°C temperature conditions. At the end of centrifugation, all of the liquid supernatant was pipetted and discarded. The bottom sample plant was analyzed again TCT determination and bound condensed tannins were detected. The extractable tannin content (ECT) was determined by the formula: ECT = TCT – BCT, in DM (Kara, 2019).

### 2.3 In vitro methane and total gas production

Rumen fluid, which is used for in vitro gas production technique, was taken from two dairy cattle (Brown Swiss). The cattle consumed approximately 65% concentrated feed mixture (cottonseed meal, soybean meal, wheat bran, DDGS, barley grain, corn bran, and corn flake) + 35% forage (wheat straw, alfalfa hay, and wet sugar beet pulp) on DM basis, by rumen tube 2 h after morning feeding. The 200±10 mg dried sample and chemical mixture (buffer + macro mineral + micro mineral + reduction + resazurin solutions) (20 mL) (Kara, 2021) and filtered rumen fluid inoculum (10 mL) in 100 mL glass syringes (Model Fortuna, Haberle Labortechnik, Germany) were incubated (Menke et al., 1979). In the study, in vitro gas production was performed with five replicates for each phonological stage. Five fermentation syringes were used as blank (without feed sample, containing only buffer, solutions, and rumen fluid) and used in the calculation as a correction value for in vitro gas production. For determination of in vitro methane production at 24 h of incubation, methane volume in 50 mL of the produced total gas) was analyzed with an infrared methane measuring device (Sensor, Europe GmbH, Erkrath, Germany). Metabolic energy (ME), the net energy of lactation (NEL), and organic matter digestion (OMd) values of forages were calculated (Menke & Steingass, 1988).

### 2.4 In vitro ruminal fermentation fluid variables

The 15 mL of in vitro fermentation fluid of all syringes at 24 h of incubation was taken to determine the concentration of straight‐short‐chain fatty acids (SCFA), branched short‐chain fatty acids (BSCFA), and ammonia‐nitrogen. The ammonia‐nitrogen concentration was determined using a commercial kit (Megazyme, Ammonia Assay Kit – Catalogue no: Product code: K‐AMIAR, Wicklow, Ireland) in a UV‐VIS spectrophotometer (SI Analytics – Xylem Analytics Germany Sales GmbH & Co. KG, Mainz, Germany). The SCFA compositions [acetic acid – AA (C2:0), propionic acid – PA (C3:0), butyric acid – BA (C4:0), valeric acid – VA (C5:0), hexanoic acid – HEXA (C6:0), heptanoic acid – HEPA (C7:0)] and BSCFA [iso‐butyric acid – IBA (C4:0i), iso‐valeric acid – IVA (C5:0i) (do not include 2‐methylbutyric acid) and iso‐caproic acid – ICA (C6:0i)] of fermentation fluid were determined in a GC‐FID (TRACE^TM^1300, Thermo Fisher Scientific, Orlando, USA). Helium was used as a carrier gas, and hydrogen was used for combustion. A polyethylene glycol column (length: 60 m, i.d.: 0.25 mm, film thickness: 0.25 μm; TG‐WAXMS, Thermo Scientific, Orlando, FL, USA) was used for this analysis in the device, and the analysis procedure was performed according to Ersahince and Kara (2017).

### 2.5 Statistical analysis

In the present study, the chemical composition and in vitro ruminal fermentation variables of pennyroyal herbage at different phenological stages were determined by one‐way analysis of variance in the SPSS 17.0 package program. When statistical significance was determined, the significance level was determined with the Tukey's multiple comparison test. The significance level was taken as *p* < 0.05. Polynomial Contrast analysis (linear and quadratic effects) revealed the effect studied variables of increasing growing stage.

## 3 RESULTS

### 3.1 Chemical composition

The chemical contents of the pennyroyal herbages used in the study are given in Table 1. As the plant phenological stages progressed, increased EE and HC values (*p* < 0.05). The CP, ADFom and NDFom values of the herbage were similar in the vegetative and seed bulking stages (*p* > 0.05), but those of full flowering stage were lower than those of other stages (*p* < 0.05). The NFC values of pennyroyal herbage at the full flowering period were higher than those of other stages (*p* < 0.05). TCT and ECT values of pennyroyal herbage were higher those of vegetative and seed bulking stages (*p* < 0.05). The BCT content for vegetative stage was higher than that of seed bulking stage (*p* < 0.05). The ash content of the herbage decreased with the progression of the phenological stage (*p* < 0.05) (Table 1). The contents of Ca, Mg, S, Zn and Fe minerals of pennyroyal herbage decreased with the increasing phenological stage (*p* < 0.05). The K, P and Na mineral levels of herbage increased with the progress in the plant growing period (*p* < 0.05), but there was no significant change in the values of Mn mineral for different phenological stages (*p* > 0.05) (Table 2).

**TABLE 1:** Chemical compositions of pennyroyal (*Mentha pulegium* L.) herbage at different phenological stages.

Phenological stages	CP	EE	Ash	ADFom	NDFom	HC	NFC	TCT	BCT	ECT
	%, DM							mg/kg DM		
Vegetative	10.38^a^	4.91^b^	10.96^a^	45.36^a^	51.64^a^	5.61^b^	22.08^b^	8.34^b^	1.83^a^	6.48^b^
Full flowering	9.89^b^	5.83^b^	11.41^a^	37.89^b^	42.80^b^	5.61^b^	30.06^a^	11.10^a^	1.40^ab^	9.69^a^
Seed bulking	11.04^a^	9.86^a^	8.69^b^	46.13^a^	55.35^a^	10.06^a^	15.03^c^	8.13^b^	1.25^b^	6.87^b^
Mean ± SD	10.44±0.34	6.87±2.34	10.35±1.27	43.13±4.23	49.93±5.91	7.09±2.23	22.39±6.86	9.19±0.98	1.50±0.30	7.68±0.96
SEM	0.21	0.78	0.42	1.41	1.97	0.74	2.28	0.66	0.10	0.65
L	0.138	<0.001	<0.001	0.622	0.090	<0.001	0.014	0.578	0.007	0.834
Q	0.048	0.013	<0.001	0.001	0.001	<0.001	0.001	0.002	0.315	0.029

Abbreviations: ADFom, acid‐free detergent‐insoluble fibrous compounds without ash; aNDFom, neutral detergent‐insoluble fibrous compounds detected by alpha amylase and without ash; BCT, bound condensed tannin; CP, crude protein; ECT, extractable condensed tannin; EE, diethyl ether extract; HC, hemicellulose (HC = NDFom – ADFom); L, linear; Mean ± SD, mean of three phenological stages ± standard deviation; NFC, nonfibrous carbohydrate; Q, quadratic; SEM, standard error of means; TCT, total condensed tannin.

^a‐c^: The difference between the means shown with different letters in the same column is statistically significant.

**TABLE 2:** Mineral composition of pennyroyal (*Mentha pulegium* L.) herbage at different phenological stages.

Phenological stages	Macro minerals, g/kg DM							Micro minerals, mg/kg DM	
	Ca	K	P	Mg	Na	S	Zn	Fe	Mn
Vegetative	18.76^a^	10.06^b^	2.47^b^	3.21^b^	0.87^b^	1.94^a^	16.05^a^	389.00^b^	47.10
Full flowering	20.31^a^	9.27^b^	2.32^b^	3.63^a^	4.63^a^	2.46^a^	25.01^a^	683.10^a^	40.05
Seed bulking	13.94^b^	13.52^a^	3.58^a^	2.58^c^	3.55^a^	0.32^b^	5.20^b^	115.12^c^	55.10
Mean ± SD	17.67±3.02	10.95±2.08	2.79±0.61	3.14±0.47	3.02±1.94	1.57±0.35	15.42±2.40	395.74±258.70	47.42±10.04
SEM	1.20	0.85	0.25	0.19	0.79	0.17	0.30	105.60	4.17
L	0.005	0.001	0.308	0.007	0.002	0.021	0.032	0.021	0.443
Quadratic	0.006	0.002	0.530	0.003	0.094	0.038	0.065	0.004	0.238

Abbreviations: L, linear; Mean ± SD, mean of three phenological stages ± standard deviation; Q, quadratic; SD, standard deviation; SEM, standard error of means. ^a‐c^, The difference between the means shown with different letters in the same column is statistically significant.

The percentages of myristic, palmitic, total saturated fatty acids (SFA) and medium‐chain fatty acids (MCFA) in the total fatty acid of the pennyroyal herbage decreased linearly with the progression of the plant phenological stage (*p* < 0.05). The percentages of oleic, ∑w‐9, medium‐chain unsaturated fatty acids (MUFA) and long‐chain fatty acids (LCFA) in the total fatty acids of the pennyroyal herbage did not change with the increasing phenological stage (*p* > 0.05). Percentages of linoleic (ω−6), α‐linolenic (ω−3), ∑ω−3, ∑ω−6, total unsaturated fatty acids (UFA) and polyunsaturated fatty acids (PUFA) in the total fatty acid of the pennyroyal herbage increased linearly with the progression of the phenological period (*p* < 0.05). The highest α‐linolenic acid level was detected in the seed bulking stage (*p* < 0.05) (Tables 3 and 4).

**TABLE 3:** Important fatty acids in total fatty acids of pennyroyal (*Mentha pulegium* L.) herbage at different phenological stages.

Phenological stages	Myristic acid	Palmitic acid	Oleic acid	Linoleic acid	α‐linolenic acid
Vegetative	0.98^a^	21.55^a^	20.73	15.38^b^	21.72^b^
Full flowering	0.83^a^	20.69^b^	25.26	21.79^a^	18.65^b^
Seed bulking	0.36^b^	14.84^c^	20.33	25.77^a^	34.56^a^
Mean ± SD	0.72±0.09	19.03±1.46	22.11±4.96	20.98±4.75	24.98±7.70
SEM	0.12	0.41	2.03	1.94	3.14
Linear	0.001	0.020	0.946	0.002	0.007
Quadratic	0.001	0.149	0.400	0.251	0.011

Abbreviations: Mean ± SD, mean of three phenological stages ± standard deviation; SEM, standard error of means; L, linear; Q, quadratic. ^a‐c^, The difference between the means shown with different letters in the same column is statistically significant.

**TABLE 4:** Saturated and unsaturated fatty acids ratio in total fatty acids of pennyroyal (*Mentha pulegium* L.) herbage at different phenological stages.

Pennyroyal herbage	SFA	UFA	MUFA	PUFA	∑ω−3	∑ω−6	∑ω−9	∑ω−3/ ∑ω−6	MCFA	LCFA	VLCFA
Vegetative	34.48^a^	65.53^b^	23.51	42.02^b^	21.99^b^	20.03^b^	21.35	1.10^ab^	0.48^a^	95.60	3.44
Full flowering	29.12^ab^	70.89^ab^	26.76	44.13^b^	19.10^b^	25.03^ab^	25.94	0.76^b^	0.28^ab^	96.87	2.84
Seed bulking	16.86^b^	83.12^a^	20.53	62.59^a^	34.68^a^	27.91^a^	20.53	1.24^a^	0.16^b^	98.25	1.49
Mean ± SD	26.82±8.42	73.18±8.41	23.60±4.69	49.58±10.22	25.26±7.58	24.32±3.57	22.61±5.00	1.04±0.23	0.31±0.15	96.91±1.38	2.59±1.11
SEM	3.44	3.43	1.91	4.17	3.09	1.46	2.04	0.09	0.06	0.56	0.46
L	0.011	0.011	0.584	0.001	0.008	0.001	0.891	0.197	0.004	0.061	0.109
Q	0.287	0.287	0.343	0.013	0.013	0.013	0.373	0.013	0.340	0.944	0.654

Abbreviations: Mean ± SD, mean of three phenological stages ± standard deviation; SEM, standard error of means; L, linear; Q, quadratic; SFA, saturated fatty acids, UFA, unsaturated fatty acids; LCFA, long‐chain fatty acids; MCFA, medium‐chain fatty acids; MUFA, monounsaturated fatty acids; PUFA, polyunsaturated fatty acids; VLCFA, very long‐chain fatty acids; ∑ω−3, total ω−3 fatty acids; ∑ω−6, total ω−6 fatty acids; ∑ ω−9, total ω−9 fatty acids. a‐b, The difference between the means shown with different letters in the same column is statistically significant.

### 3.2 In vitro ruminal fermentation, ammonia‐nitrogen and organic acid values

In vitro 24‐h total gas production, methane production and OMd of the pennyroyal herbage decreased linearly with the progression of the phenological stage (*p* < 0.05). However, ME and NEL values of forages at full flowering stage were lower than those of vegetative and seed bulking stages (*p* < 0.05). The ME value of the herbage was similar in the seed bulking and the vegetative stages, and the NEL value in the seed bulking stages was higher than the vegetative and full flowering stages (*p* < 0.05).

Concentration of ammonia‐nitrogen in the in vitro ruminal fermentation fluid of the pennyroyal herbage at the seed bulking stage was lower than those of other phenological stages (*p* < 0.05) (Table 5). Concentrations of straight‐short‐chain fatty acids (SCFA) (acetic acid (AA), propionic acid (PA), and valeric acid (VA)), branched short chain fatty acid (BSCFA) (iso‐caproic acid (ICA), iso‐butyric acid (IBA), iso‐valeric acid (IVA)), hexanoic acid and heptanoic acid in the in vitro ruminal fermentation liquid of the pennyroyal herbage were similar for different phenological periods (*p* > 0.05). However, molarities of total short chain fatty acid (T‐SCFA) and butyric acid (BA) in the in vitro ruminal fermentation liquid of the pennyroyal herbage at full flowering stage were lower than those of vegetative and seed bulking stages (*p* < 0.05) (Table 6).

**TABLE 5:** In vitro rumen fermentation and ammonia‐N value of pennyroyal (*Mentha pulegium* L.) herbage at different phenological stages.

Phenological stages	Total Gas	Methane	ME	NE_L_	OMd	Ammonia‐N
Vegetative	33.84^a^	4.84^a^	12.53^a^	9.05^b^	56.49^a^	90.44^a^
Full flowering	31.15^b^	4.47^b^	11.36^b^	8.07^c^	53.69^b^	95.34^a^
Seed bulking	29.75^b^	4.21^b^	12.40^a^	9.78^a^	51.71^c^	79.66^b^
Mean ± SD	31.58±2.03	4.51±0.39	12.10±0.56	8.97±0.74	53.97±2.22	88.48±7.68
SEM	0.52	0.10	0.14	0.19	0.57	2.56
Linear	<0.001	0.007	0.196	<0.001	<0.001	0.006
Quadratic	0.307	0.758	<0.001	<0.001	0.455	0.013

Abbreviations: Ammonia‐N, ammonia‐nitrogen as g/L, fermentation fluid; ME, metabolic energy calculated from in vitro total gas production, as MJ/kg DM; Mean ± SD, mean of three phenological stages ± standard deviation; Methane, in vitro methane gas volume (mL) produced for 0.2 g DM at 24 h; NE_L_, net energy of lactation for dairy cattle, calculated from in vitro total gas production, as MJ/kg DM; OMd, organic matter digestion, calculated from in vitro total gas production at 24 h ruminal incubation; SEM, standard error of means; Total Gas, in vitro total gas volume (mL) produced for 0.2 g DM at 24 h.

**TABLE 6:** SCFA and BSCFA concentrations in the in vitro rumen fermentation fluid of pennyroyal (*Mentha pulegium* L.) herbage at different phenological stages (mmol/L rumen fluid).

Phenological stage	T‐SCFA	AA	PA	BA	VA	HEXA	HEPA	IVA	IBA	ICA
		SCFA				OA		BSCFA		
Vegetative	71.64^a^	51.81	11.34	5.75^a^	0.62	0.36	0.25	0.70	0.56	0.24
Full flowering	65.09^b^	47.49	10.13	4.84^b^	0.60	0.34	0.25	0.66	0.53	0.24
Seed bulking	69.14^a^	50.51	10.62	5.32^a^	0.62	0.35	0.25	0.68	0.55	0.24
Mean ± SD	68.62±3.41	49.94±3.16	10.70±0.77	5.30±0.46	0.61±0.02	0.35±0.01	0.25±0.01	0.68±0.03	0.55±0.02	0.24±0.001
SEM	1.47	1.05	0.26	0.15	0.01	0.01	0.01	0.01	0.01	0.001
L	0.460	0.600	0.220	0.100	0.910	0.290	0.710	0.410	0.590	0.420
Q	0.010	0.120	0.110	0.010	0.170	0.180	0.310	0.140	0.300	0.180

Abbreviations: BSCFA, branched short‐chain fatty acid = ICA + IBA + IVA; T‐SCFA, total short‐chain fatty acid = SCFA + BSCFA; HEPA, heptanoic acid; HEXA, hexanoic acid; IBA, iso‐butyric acid; ICA, iso‐caproic acid; IVA, iso‐valeric acid; L, linear; Q, quadratic; SCFA, straight‐short‐chain fatty acids = AA + PA + BA + VA + HEXA + HEPA; SD, standard deviation; SEM, standard error of means.

## 4 DISCUSSION

### 4.1 Chemical content

As an herbivore, ruminants should have fibre‐containing feedstuffs in their rations. The pennyroyal plant, which grows naturally in pasture areas, has similar macronutrients to traditional forages and also contains aromatic volatile compounds. In the present study, the fiber compounds (such as NDFom and ADFom) of pennyroyal herbage differed with increased plant growth. As the plant phenological period progresses in cultivated forage plants, it is expected that the content of fibrous compounds will increase (Kara et al., 2018). During the plant phenological stage, the increase in the hemicellulose content and the significant decrease in the NFC value at the seed bulking stage may negatively affect the ruminal fermentation of the plant. The lower in vitro gas production at the seed bulking stage in the study compared to other periods can be related to the hemicelluloses and lignin covalently cross‐linking could increase with the plant growing stage (Terret & Dupree, 2019). The CP levels of pennyroyal herbages for three phenological stages were approximately 10–11%, indicating that the forage had a moderate CP content for ruminants (NRC, 2001). Aziz et al. (2019) stated that pennyroyal herbage included 10.94% CP (1.75% nitrogen × 6.25).

As the plant development period of the forage crops progresses, the mineral content decreases. It was determined that the EE content of the plant increased according to the plant phenological stage and reached 9% in DM. The high EE content during the seed bulking stage was predicted to originate from the plant seeds. However, it has been estimated that the EE content of about 5% and 6% in the vegetative and full flowering stages of the plant, respectively, is due to the fact that this plant is drought tolerant and collects waxy compounds in its leaves to prevent water loss. In another study, it was reported that the pennyroyal plant contains 3.3% oil (Selmi et al., 2022). Considering that the cultivated forage plants contain 2%–3% EE (NRC, 2001), it shows that pennyroyal plant will be a feedstuff or feed additive with its high oil and high ∑ω−3 and ∑ω−6 fatty acids as a functional rumen modulator (Ebrahimi et al., 2017; Fiorentini et al., 2015; Roy et al., 2017).

In the present study, major fatty acids of pennyroyal herbage for different phenological stages were α‐linolenic, linoleic, oleic and palmitic acids, respectively. These fatty acids constituted about 88% of fatty acids in total fatty acids of pennyroyal herbage. Maffei and Scannerini (1992) found a high level of α‐linolenic only in the leaves of certain *Mentha* species (*M. longifolia*, *M. crispa*, and *M. sachalinensis*). Among the major components found in peppermint leaves are fatty acids such as linoleic, α‐linolenic and palmitic acids (Pérez et al., 2014). The ω−6 linoleic acid and ω−3 α‐linolenic acid contents of pennyroyal herbage increased with phenological stage may be related to the fatty acid content of plant flower or seed parts. The increasing plant’ growth stages decreased UFA myristic and palmitic acids percentages in total fatty acids of herbage demonstrated that pennyroyal herbage flowering and seed bulking stages were functional (rich essential fatty acids such as ω−3 α‐linolenic acid and ω−6 linoleic acid) properties in terms of fatty acids. In addition, the highest α‐linolenic acid level in the post‐flowering period suggests that using the herbage as a forage source during the seed bulking stage will have a positive effect on rumen fermentation. In cultured forage plants, as the plant phenological period progresses, the ratio of α‐linolenic (ω−3) decreases and the ratio of oleic acid and UFA fatty acids increases (Glasser et al., 2013). As the phenological stage of the pennyroyal herbage, the increase in the ratios of α‐linolenic (ω−3) and linoleic (ω−6) acids increased the value of the herbage in terms of essential fatty acids with the progression of the phenological period, and 5%–10% EE value in pennyroyal herbage is higher than those of traditionally used cultivated meadow‐pasture plants (Dierking et al., 2010; Glasser et al., 2013; NRC, 2001).

In the present study, the ash level of the pennyroyal plant decreased from approximately 11%–8.7% with the progression of the phenological stage. Previous researchers have shown that Ca, K, Mg, Fe, and Mn levels of pennyroyal herbage, collected from the wild populations, according to the geographical region where the plant is collected, are consistent with the findings of the present study (Mohammadi & Asadi‐Gharneh, 2018). For this plant, Ca, Mg, and S, which are macro minerals, and Fe and Zn, which are micro minerals, decreased with plant growth, but K, P, and Na levels increased. The levels of K, Mg, minerals detected for pennyroyal herbage in the present study, were similar to the results of a previous study (Aziz et al., 2019), but Ca and P contents were lower than the results of a previous study (Aziz et al., 2019) and Fe and Mn levels were higher than the results of previous study (Aziz et al., 2019; Selmi et al., 2022). The Ca and Mg contents of pennyroyal herbage were higher than those of Selmi et al. (2022). This shows that the addition of the pennyroyal herbage to the ration as forage was a good source of Ca from macro minerals; it could be preferred as a source of Ca in the ration when adjusting the Ca/P ratio, and it was lower than legume and grasses’ forages in terms of cationic K mineral (Kara et al., 2018; NRC, 2001). Although the Fe mineral was a mineral that is high in pennyroyal herbage at vegetative and full flowering stages, forages obtained in these phenological stages could be met during the growth period of calves (NRC, 2001). The Fe mineral concentrations of the pennyroyal herbage in the full flowering and vegetative stages were higher than those of a widely used cultured grass (ryegrass, timothy, Bermuda grass, orchard grass, red fescue, and smooth brome) and legume (red clover, white clover, and alsike clover) plants, and it showed that this plant could be an important Fe source (Juknevičius & Sabienė, 2007; NRC, 2001). Therefore, the Fe content of pennyroyal herbage was lower than that of some legumes (*Medicago sativa* and *Vicia sativa*) and grasses (*Brachiaria mutica* and *Panicum maximum*) herbages (Melesse et al., 2017; NRC, 2001).

### 4.2 In vitro ruminal fermentation

Due to global warming, the production of forage crops and the permanence of green grass in pasture areas throughout the summer has decreased (Kara et al., 2015). Even if the forage quality is not very high, attention is drawn to the usability and functional properties of medium‐quality alternative plants as forage. *Mentha* species are drought‐resistant plants and have a very wide distribution in the world (Franz Vienna et al., 2007). No study has been found on the in vitro ruminal fermentation of the pennyroyal plant. In this study, even though in different phenological stages, this plant had in vitro digestibility values of medium‐ to good‐quality forage. Although the in vitro 24‐h total gas production, methane production, and OMd of the pennyroyal herbage decreased linearly with the progress of the phenological stage, it was understood that the ME and NE_L_ values calculated by the nutrient composition of the plant were at higher levels in the full flowering period compared to the vegetative stage. The concentrations of T‐SCFA and SCFA such as AA, PA, and BA in the in vitro ruminal fermentation fluid of the pennyroyal herbage are an indicator of the in vitro fermentation capacity, and the fact that they did not change with the progress in the phenological stage in this forage plant is consistent with the results of ME and NEL. The molarities of acids such as AA, BA, PA, and VA, which are the end products of carbohydrate catabolism in rumen fluid, in the in vitro fermentation fluid were at reference values (Kara et al., 2018). Besides, the higher concentration of BA, which is also produced by amino acid fermentation (Li et al., 2020), in the in vitro fermentation fluid for samples at vegetative and seed bulking stages is due in part to protein degradation. Among these acids, the molarity of BA in the in vitro fermentation fluid is higher in the vegetative and seed bulking stages than in the full flowering stage, which may be related to TCT and ECT contents. According to the references, the TCT level of the plant was at a level that could reduce ruminal methane production. Ruminal protein degradation may also be adversely affected by >2%–3% TCT in plants (Kara, 2019; Min et al., 2006). However, with medium‐low TCT content in this plant, it caused a significant decrease in ruminal BA concentration (as well as a numerical decrease in acetic acid) and methane production during the full flowering period and low methane production during the seed setting period as a result of low ruminal fermentation. It was observed that ME and NEL values calculated from in vitro gas production values were lower in the full bloom period than in both periods. Methane gas has an undesirable positive effect on global warming (IPCC, 2014). For this reason, it is important to use alternative forage that reduces methane production in ruminant feeding. The decrease in the in vitro total methane emission from the pennyroyal plant fermentation with the progression of the plant phenological stages showed that the plant was anti‐methanogenic in the later phenological periods. Low methane production at the seed bulking stage may come forth less in vitro fermentation (low total gas production) due to related to the increased lignin hemicellulose complex of the plant (Terrett & Dupree, 2019). Besides, low methane production at the seed bulking stage can be connected to the anti‐methanogenic property of the high level of oil content and ω−3 and ω−6 fatty acids at the seed bulking stage (Yilmaz and Kara, 2022b; Sun et al., 2022).

In a previous study, it was determined that the addition of dried mint plant (*Mentha piperita* L.) to the dairy cattle ration at different rates decreased in vitro ruminal methane production, ammonia production, the count of total bacteria, and number of *Entodiniomorpha* but increased in vitro total gas production and did not affect in vitro ruminal DM digestion (Zmora et al., 2012). Another study showed a positive effect on digestibility by feeding peppermint. The authors concluded that peppermint had great potential as a natural manipulator of rumen fermentation of beef cattle by depressing ammonia‐nitrogen concentration or numbers of protozoa (Ando et al., 2003). Although there was no difference in the concentrations of iso‐acid (such as iso‐butyric, iso‐valeric and iso‐caproic acids), which are a by‐product of ruminal protein catabolism, for the three phenological stages, the ruminal ammonia‐nitrogen concentration in the seed bulking stage was lower than in other stages can be related to the decreased ruminal protein catabolism due to the increase in the fiber‐nitrogen complex ratio or TCT in this stage (Kara et al., 2022a; Kara, 2019). A previous study reported that increasing additions of mint essential oil, which included 39.98% pulegone and 38.99% α‐pinene, to the total mix ration linearly reduced in vitro ruminal methane production (Rofiq et al., 2021). Besides all these positive results, pennyroyal has a traditional folk medicine use in inducing abortions. These oils are high in pulegone, a highly toxic volatile, which can stimulate uterine activity (Franz Vienna et al., 2007). The pulegone, which is monoterpenoid, level of the pennyroyal plant was not analyzed in the present study. There is no information in the literature about the absorbability of pulegone in ruminants. Previous a study reported that the essence of *Mentha longofolia*, which contained pulegone (17.3%), profoundly altered gastrointestinal smooth muscle contraction in a dose‐dependent and tissue‐specific manner and had the potential for development as a prokinetic and relaxant agent that may prevent or alleviate dysfunctions of gastrointestinal motility (Jalilzadeh‐Amin et al., 2012). In addition, Geiger et al. (2021) determined that menthol, one of the aromatic compounds of pennyroyal, stimulated Ca^2+^ absorption in ruminants. Researchers stated that menthol could be used as a tool to enhance ruminal Ca^2+^ absorption and to prevent hypocalcemia in dairy cows (Geiger et al., 2021).

As a result, the pennyroyal herbage is a functional plant with a high percentage of EE, α‐linolenic acid, linoleic acid, and  ∑ω−3 fatty acids, and Ca, Fe, and Zn minerals. The in vitro ruminal fermentation level and nutrient composition of this plant indicate that it will be a good‐quality forage, especially today when the effect of global warming is evident. However, these results need to be supported by in vivo feeding trials.

## AUTHOR CONTRIBUTIONS

Conceptualisation: KK. Methodology: KK. Formal analysis and investigation: KK, SY, SD, BKG. Writing – original draft preparation: KK. Writing – review and editing: KK. Resources: KK, SY, BKG. Supervision: KK. All authors reviewed the manuscript.

## CONFLICT OF INTEREST STATEMENT

The authors declare that they have no potential conflicts of interest with respect to the research, authorship and/or publication of this article.

## FUNDING INFORMATION

This study was supported by the Research Fund of Erciyes University (Kayseri, Türkiye) with Project ID: TSA‐2023‐12665.

## ETHICS STATEMENT

This study has an in vitro procedure; therefore, ethics committee approval is not required.

The authors confirm that the ethical policies of the journal, as noted on the journal's author guidelines page, have been adhered to, and the appropriate ethical review committee approval was received.

## CONSENT FOR PUBLICATION

All authors have consented to the publication and presentation of the data in this article.

### PEER REVIEW

The peer review history for this article is available at https://publons.com/publon/10.1002/vms3.1397.

## Data Availability

The data analysed in this investigation are available upon request to the corresponding author.
